# Functionalized Porous Silica-Based Nano/Micro Particles for Environmental Remediation of Hazard Ions

**DOI:** 10.3390/nano9020247

**Published:** 2019-02-12

**Authors:** Chun Min Li, Xin Peng Wang, Zi Hao Jiao, Yu Sheng Zhang, Xiang Biao Yin, Xue Min Cui, Yue Zhou Wei

**Affiliations:** 1 School of Resources, Environment and Materials, Guangxi Key Laboratory of Processing for Non-ferrous Metallic and Featured Materials, Guangxi University, Nanning 530004, China; 15296576429@163.com (C.M.L.); jzh923174679@163.com (Z.H.J.); 15676170158@163.com (Y.S.Z.); yzwei@gxu.edu.cn (Y.Z.W.); 2 Laboratory for Advanced Nuclear Energy, Tokyo Institute of Technology, 2-12-1, Ookayama, Meguro-ku, Tokyo 152-8550, Japan; yin.x.aa@m.titech.ac.jp; 3 School of Chemistry and Chemical Engineering, Guangxi University, Nanning 530004, China; cui-xm@tsinghua.edu.cn

**Keywords:** silica-based nano/microparticles, inorganic materials, organic groups, adsorption, hazard ions

## Abstract

The adsorption and separation of hazard metal ions, radioactive nuclides, or minor actinides from wastewater and high-level radioactive waste liquids using functional silica-based nano/micro-particles modified with various inorganic materials or organic groups, has attracted significant attention since the discovery of ordered mesoporous silica-based substrates. Focusing on inorganic and organic modified materials, the synthesis methods and sorption performances for specific ions in aqueous solutions are summarized in this review. Three modification methods for silica-based particles, the direct synthesis method, wetness impregnation method, and layer-by-layer (LBL) deposition, are usually adopted to load inorganic material onto silica-based particles, while the wetness impregnation method is currently used for the preparation of functional silica-based particles modified with organic groups. Generally, the specific synthesis method is employed based on the properties of the loading materials and the silicon-based substrate. Adsorption of specific toxic ions onto modified silica-based particles depends on the properties of the loaded material. The silicon matrix only changes the thermodynamic and mechanical properties of the material, such as the abrasive resistance, dispersibility, and radiation resistance. In this paper, inorganic loads, such as metal phosphates, molybdophosphate, titanate-based materials, and hydrotalcite, in addition to organic loads, such as 1,3-[(2,4-diethylheptylethoxy)oxy]-2,4-crown-6-Calix{4}arene (Calix {4}) arene-R14 and functional 2,6-bis-(5,6-dialkyl-1,2,4-triazin-3-yl)-pyridines(BTP) are reviewed. More specifically, we emphasize on the synthesis methods of such materials, their structures in relation to their capacities, their selectivities for trapping specific ions from either single or multi-component aqueous solutions, and the possible retention mechanisms. Potential candidates for remediation uses are selected based on their sorption capacities and distribution coefficients for target cations and the pH window for an optimum cation capture.

## 1. Introduction

Functionalized silica-based nanoparticle materials have opened a wide range of opportunities in research fields related to catalysis, separation, and adsorption [1,2] since the discovery of the ordered mesoporous silica-based carriers, such as MCM-41, MCM-48, M41S [3,4,5], SBA-1 [6], SBA-15 [7], FSM-16 [8], and silica spheres [9]. These materials have mesoporous channels, high porosities, and large specific surface areas (BET). Furthermore, their narrow distributions of pore sizes, and shapes can be controlled by choosing different structural directing agents [10,11,12,13]. Important indices of common silica-based carriers are listed in Table 1. Most silica-based mesoporous materials do not have specific surface properties, which limits their applications in fields such as ion exchange, catalysis, sensing, and adsorption, which require stereochemical configurations or charge densities, specific binding sites, and acidities [14,15]. However, the sufficiently large pore sizes of mesoporous silica can accommodate various large molecules. Furthermore, a variety of functional silica-based materials can be synthesized due to the high density of silanol groups in the pore channels, which can be used to introduce functional groups with a high coverage rate [16]. A wide variety of materials with specific surface properties have been loaded onto silica-based particles, and applied in many areas of science and technology [15]. In particular, functional silica nanoparticles are exploited to produce a photoacoustic signal enhancement in the biological window for molecular and cellular characterization of cancer through near-infrared (NIR)-absorbing dyes or organic/inorganic nanoparticles [17]. This present review is concerned with synthesis processes of functional silica-based particles modified using various inorganic or organic materials, and their applications in the adsorption of environmental wastewater that contains typical radioactive ions and hazardous heavy metal ions.

Methods to effectively link a variety of specific inorganic adsorption species, or organic chelating ligands, onto the surface of silica-based carriers are key for realizing various adsorption applications. The layer-by-layer deposition method was reported more than ten years ago. This method allows the growth of ultrathin films on the surface or walls of silica-based particles [18,19,20]. Mineral adsorption materials with bidimensional-layered structures, such as zirconium and titanium phosphonates, are easily deposited onto silica-based carriers [21,22]. Indeed, this method has been successfully generalized for complex oxides, such as niobates [23] or perovskites [24]. 

**Figure 1:**
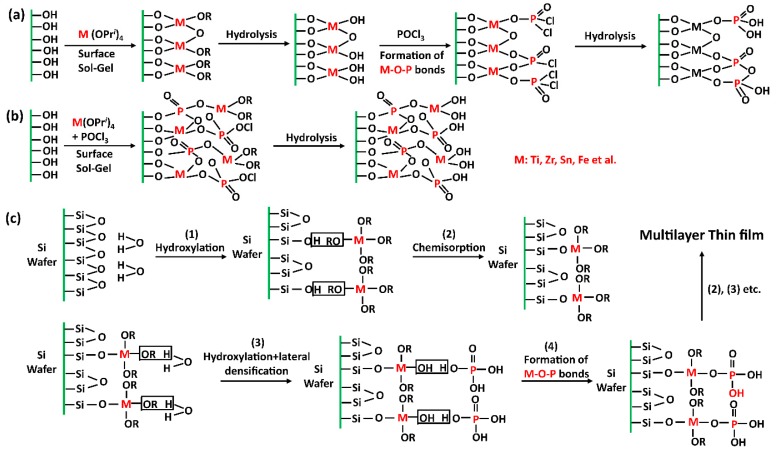
(**a**) Modification of SBA-15 surfaces by alternate grafting with M (OPri)_4_ and POCl_3_ (Method 1); (**b**) modification of SBA-15 surfaces by one-pot grafting of metal phosphate formed in situ (Method 2); and (**c**) principle of a sequential deposition of a metal phosphate [31].

Ionic layer deposition is similar to the layer-by-layer deposition method, and extends this approach for the deposition of a large variety of oxides, such as hydroxides or sulfides [25]. Furthermore, materials with heterostructures, based on alternate layer deposition of ionic colloidal species, have also been reported [26,27,28,29]. The new “surface sol–gel” method is also derived from this layer-by-layer deposition method, using sequential chemisorption and activation steps, and it allows better control of film deposition at a molecular level. The linking mechanism of the layer-by-layer deposition method is based on the reactions between acid group anions and the metal oxide surface, as well as the feasibility of metal salt with acid group anions [21,22,30]. The specific grafting mechanism of acid group anion reactions, with metal oxide, onto the surface of silicon oxide is presented in Figure 1. 

The aqueous phase impregnation method is another effective way for immobilizing specific inorganic adsorbents onto silica-based nanoparticles [32,33,34]. This surface impregnation method allows better control over the adsorbent’s homogeneity. Generally, an impregnation-calcination -activation process is required in this method [33,34]. The synthesis procedure and parameters are key for loading specific substances onto the silica-based substrate. As most inorganic adsorbents are insoluble in aqueous solutions, one-step impregnation is obviously infeasible because there are no significant attractive forces between the adsorbent and silica-based substrate [35]. In this method, acid radical groups are first immobilized inside the channel of the silica-based particles through an acid–base interaction between the heteropoly acid and silanol groups [36,37], and one proton from the acid radical group interacts with Si-OH, producing the SiOH^2+^ group, which serves as a counteractive site for metal ions. This chemical reaction produces a stable and non-physical adsorption pattern for adsorption intercalation within the substrate [38], and dissolution during the adsorption process is prevented. It is important to carefully design the heating rate to prevent adsorbent aggregation on the silica-based substrate, and the dissolution during the adsorption procedure [34,35].

**Figure 2:**
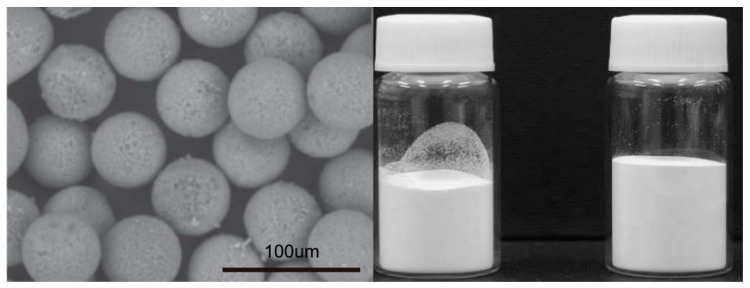
SiO_2_-P and SiO_2_-P modified with organic groups [48].

The layer-by-layer deposition and surface impregnation methods are available for many inorganic adsorbents immobilized on silica-based particles, and effectively remove the specific environmental hazardous ions in aqueous solution. We summarized the silica-based materials modified by the main inorganic material in Table 2. These methods may be unsuitable for immobilizing organic adsorbents on the surface of silicon. Reports to date have shown that silica-based nanoparticles modified via organic ligand groups, not only can be used as catalysts or highly effective adsorbents for the removal of typical radioactive and metal ions from wastewater [39,40,41,42,43], but also significantly improve the hydrothermal and mechanical stability of the organic adsorbent [44]. Therefore, organic-modified silica-based nanoparticles possess the advantage of inherent stability and show promise for advanced applications. In addition, organic ligands offer the flexibility of choosing the functional modifier to remove specific ions, and are of suitable sizes for modifying desired structural properties of the silica-based carrier. At the moment, modification of a silica-based substrate with organic ligands is always achieved via surface modification or direct synthesis. Recently, a new type of porous silica-based microsphere was developed by Wei’s team [45,46,47], and Figure 2 shows the scanning electron microscope (SEM)and pictures of real silica sphere products. Particle size, mean pore size, and pore fraction of this new type of sphere were 40–60 µm, 600 nm, and 0.69, respectively. Silica spheres modified by a polymer (SiO_2_-P) can also be synthesized for the purpose of loading various organic adsorbents onto the substrate. The SiO_2_-P inorganic/organic hybrid support is prepared by impregnating a copolymer, such as styrene-divinylbenzene copolymers, into a microporous SiO_2_ substrate. SiO_2_-P substrates have been successfully modified by many organic macromolecular recognition materials. Table 3 summarizes the SiO_2_-P adsorbents modified with the specific organic macromolecules and their applications for treating various ions.

Sorption-based technologies used for heavy metal removal require suitable sorbents for retention of radioactive ions from aqueous streams [61]. However, retention mechanisms in a given system are not always suitable for other systems or experimental conditions. Heavy metals could be removed from aqueous solutions in the form of complex cations, free cations, or free oxyanions, through an ion-exchange mechanism or by the formation of precipitates at the interface as hydroxides or insoluble oxides. The removal style depends on the surface properties of the adsorption materials, metal nature, and the environment of the aqueous solution [62,63,64,65,66,67,68,69,70,71]. In this regard, the materials fabricated for radioactive ion removal are based on materials designed for heavy metal removal. For example, single-component sorption of Sr^2+^ ions onto inorganic adsorbents occurs due to the outer-sphere surface complexes, and the adsorption of cesium cations on aluminosilicate surfaces occurs due to outer-sphere surface complexes [63,72,73,74]. However, the treatment of radioactive ions using sorption-based removal requires specificity that is not required for heavy metal removal. When exposed to a high-radiation environment, the adsorption capacity and adsorbents’ selectivity can be significantly altered. Generally, inorganic adsorbents are more resistant to radiation degradation compared to organic ion-exchangers or adsorbents [75]. For organic polymer adsorbents, changes in the cross-linking and main chain scission are the most important chemical effects when in a high-radiation environment [76]. This is due to the degradation of the eventual formation of low-molecular organic complexants, which increases the mobility and solubility of the radionuclide and decreases the retention properties. Furthermore, thermal explosion accidents have also been reported when ion-exchange resins were applied in the presence of nitric acid and strong oxidants solution [77]. Nevertheless, mesoporous silica-based materials modified by functional organic macromolecules and resin, which will be discussed in this review, exhibit high radiolytic stabilities.

Recently developed functional silica-based adsorbents are reviewed here with an emphasis on surface functionalization methods, their structures in relation to their capacities, selectivity of target ions from either single or multi-component aqueous solutions, and the possible mechanisms of their adsorption. Compared to traditional adsorption materials, functional silica-based adsorbents simultaneously possess properties such as structural flexibilities, thermal stabilities, high mechanical strengths, strong acid and radiation resistances, facile solid-liquid separation, and affinities for target cations. Furthermore, taking advantage of the synergy between these particular functional materials and silica-based substrates would remarkably boost functional adsorbents for practical applications.

## 2. Porous Silica-Based Nanoparticles Modified by Inorganic Materials

### 2.1. Silica-Based Nanoparticles Modified by Metal Phosphates

Metal phosphates have been discovered over the last century, and studies on layered metal phosphates and their derivatives began in the early 1950s. Later studies found that some of these salts could effectively remove radioactive ions in radioactive wastes streams [27]. Tetravalent metal phosphates have extremely low solubilities, and these phosphates were amorphous initially. However, in 1964, Clearfield and Stynes first synthesized the crystalline phosphatic compound, which elucidated the structure and chemical reactivity of phosphates [78]. In the field of inorganic ion-exchange, layered-type metal phosphates have been widely studied and applied [79,80]. Many layered metal phosphates and open-frameworks have been developed owing to their various compositions and properties [81,82,83,84]. Nevertheless, the lack of a large surface area and small pores or interlayer spaces severely constrains their applications. There has been considerable research on effectively utilizing the excellent properties of metal phosphates in various applications, especially in sorption. Generally, all these research efforts aimed at loading metal phosphates onto a reliable substrate via physical or chemical methods. Among these substrates, silica-based nanoparticles are usually the best choice. In this section, the synthesis procedure, application of sorption of ions, and sorption mechanisms of functional silica-based nanoparticles modified with metal phosphates are discussed.

#### 2.1.1. Direct Synthesis of Mesoporous Metal Phosphates via Surfactant Templating

The method of directly synthesizing porous metal phosphates usually uses surfactant templating. For this purpose, a metal (such as zirconium, titanium, or their mixtures) and cationic surfactant are first dissolved in deionized water by heating at a proper temperature. Although the order of components addition may vary in different synthesis processes [85,86], the purpose is always to achieve homogeneous mixing between the metal ions and surfactant templating. The required amount of H_3_PO_4_ is added dropwise to the mixture. A gel is homogeneously formed with vigorous stirring for a specified amount of time. Finally, the gel is stirred at a specific temperature for a long period. The product is obtained by filtration, washed with deionized water, and dried under a vacuum. There are various methods for removal of the surfactant template, such as direct calcination, solvent extraction in ethanol/HCl, and solvent extraction. The mechanism of the synthesis is presented in Figure 1a.

#### 2.1.2. Synthesis of Functional Silica-Based Nanoparticle with Metal Phosphates via Wetness Impregnation

Typically, a mixed solution of metal salts and phosphoric acid is first immersed with silica-based nanoparticles, forming a suspension. Subsequently, the suspension is moved into a vacuum rotary system for a specified amount of time to ensure the mixed metal ions sufficiently fill the channels of the substrate. After drying in the vacuum rotary system, the dried powder is finally calcined at a specified temperature to obtain the desired product on the surface of the substrate [33,49].

#### 2.1.3. Synthesis of Functional Silica-Based Nanoparticle with Metal Phosphates via a Layer-by-Layer Method

The layer-by-layer (LBL) deposition approach is based on solution phases. This method is generally used to simply prepare ultrathin and multilayer films with interlayer distance variability and layer interpenetration inevitability. Typically, during the synthesis of silica-based nanoparticles modified with titanium phosphate, the silica-based nanoparticles are added to a two-neck flask by injecting anhydrous toluene and Ti(OPri)_4_ at room temperature. The slurry is refluxed and stirred for 2 h, after which it is filtered and washed three times with anhydrous toluene, and three times with deionized water for hydrolysis. For each washing process, 30 mL of anhydrous toluene and deionized water are added to a centrifuge tube, a vortex is used to disperse the solid, the suspension is centrifuged, and finally, the supernatant is discarded. The sample is dried in air at 80 °C for 24 h, then POCl_3_ is added to the sample using the same procedure, and a reaction with POCl_3_ occurs in place of the Ti(OPri)_4_. A silica-based nanoparticle-Ti-P-Ti-P product is obtained [49]. Other metal phosphates have a similar synthesis process and the mechanism of synthesis is presented in Figure 1c.

#### 2.1.4. Adsorption Performance of Ion Sorption

Metal phosphates and silica-based nanoparticles modified with metal phosphates have been widely used as ion exchangers due to the negative charges of the surface hydrophosphate groups [87,88,89,90]. For instance, lanthanum phosphate exhibits a high adsorption capacity of metal ions in aqueous solutions [50]. The adsorption of Cr (III), Mn (II), Fe (III), Co (II), Ni (II), Cu (II), Zn (II), Cd (II), Ba (II), Hg (II), and Pb (II) lanthanum phosphate samples has been studied, and the results show that mercury and nickel ions are highly adsorbed onto lanthanum phosphate compared to other ions. The sorption results are listed in Table 4. In an ammonia solution, the adsorption of Cr (III), Mn (II), Fe (III), Co (II), Ni (II), Cu (II), Zn (II), Cd (II), Ba (II), Hg (II), and Pb (II) increases due to the formation of ammonia complexes [51], which enhance their sorption interactions with the adsorbent (Table 5). The adsorption of Ni^2+^ and Hg^2+^ ions significantly decreased in potassium ferrocyanide and potassium ferricyanide solutions. The charges and strengths of the complexes between metal ions and cyanide, and the pore sizes of channels of the adsorbents may limit the adsorption capacities for the metal ions [91]. Titanium phosphate materials, which have large surface areas, also possess the ion-exchange ability for metal ions. The adsorption of Pb^2+^ on the adsorbent of silica-based-Ti-P can reach up to 0.45 mmol/g, and these materials show much higher sorption capacities with a high content of titanium phosphate. The experimental results have demonstrated that silica-based-nanoparticles modified with metal phosphate exhibit higher adsorption of metal ions than silica-based-nanoparticles and metal phosphate [31]. The value of the distribution coefficient (Kd) controls the adsorption of each species on an adsorbent. Similar elements or species can be adsorbed onto the adsorbent through selective removal and separation of the species of toxic metal ions in aqueous solutions. Therefore, the selective removal of hazardous ions in aqueous solutions requires new selective methods that can be inspired by new adsorbents with various porous frameworks.

#### 2.1.5. Specific Retention Mechanism of Ion Sorption

Silica-based nanoparticles modified with metal phosphate composites exhibit ion-exchange properties attributed to the defective P-OH groups. O-H bonding in silanols is absolutely covalent, while O-H bonds in P-OH^+^ possess ionic behavior for ion-exchange capacity [86]. Furthermore, the framework phosphonium cations simultaneously increase the anion exchange capacity. Along with the anion exchange property, a cation exchange property was observed for TCM-7/-8 due to the defective P-OH. Unlike silanols (Si-OH), in which O-H bonding is purely covalent, the high ionic character of the O-H bond in the P-O-H^+^ is responsible for the cation exchange capacity. Ti and P have regular alternating tetrahedral arrangements. This model explains the anion and cation exchange capacities of these materials [85].

### 2.2. Silica-Based Nanoparticles Modified with Molybdophosphate

**Figure 3:**
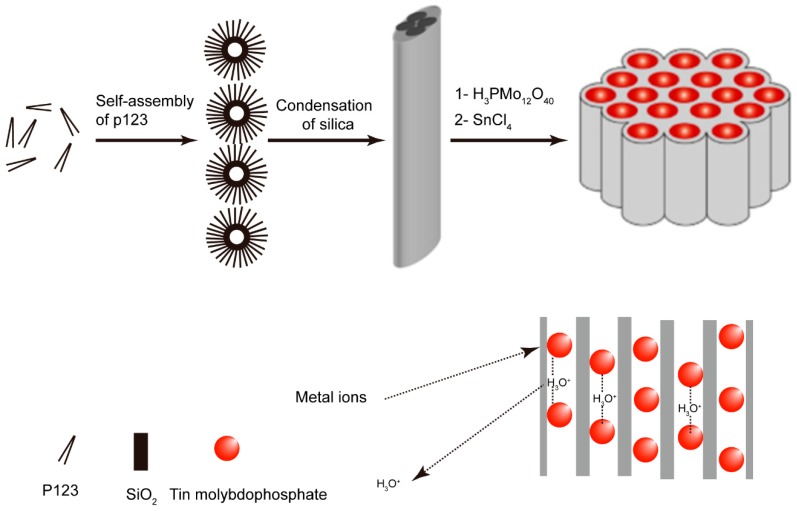
Schematic diagram of the proposed synthesis procedure for immobilization of TMP onto mesoporous silica SBA-15, and the ion-exchange process [32].

The metal molybdophosphate (MMP) has attracted considerable attention based on its high specificity adsorption of ions and stability in acidic environments [92]. Nevertheless, the small particle size (1–5 µm [93]) of MMP microcrystalline structure is impervious to liquids, preventing its use in column processes in pure form. A promising method to realize the full use of MMP is to immobilize it and insert supporting materials. Supporting materials such as zirconium phosphate [94], polymeric resins [95], calcium alginate [96], and polyacrylonitrile (PAN) [97] have been adopted to engineer MMP’s into an acceptable form for column applications. The method of immobilizing MMP’s into the silica-based nanoparticles through a sol–gel process is promising, and offers the possibility of immobilizing MMP’s onto silica matrices to provide a product with adequate surface area. Furthermore, the simple aqueous phase impregnation method is another effective method of immobilizing metal molybdophosphate onto silica-based substrates, which was adopted for the preparation of Tin (IV) molybdophosphate (TMP) onto mesoporous silica SBA-15 [32]. The schematic diagram of the proposed synthesis procedure for immobilization of Tin (IV) molybdophosphate (TMP) onto mesoporous silica SBA-15 and ion-exchange process is shown in Figure 3.

#### 2.2.1. Synthesis of Functional Silica-Based Nanoparticle with Metal Phosphomolybdate via a Crystallization Sol-Gel Method

Silica-based nanoparticles modified with ammonium molybdophosphate (AMP) are usually prepared by a solution crystallization sol-gel method [98]. First, tetramethylorthosilicate (TMOS) is added to a methanol solution with the molar ratio of 1:12 to form a silica gel. Second, various quantities of AMP are dissolved in 10 M ammonium hydroxide to obtain the final composites containing different mass percentages of ammonium molybdophosphate. This solution is added to the TMOS-methanol mixture, transforming it into a clear gel within 20 min. Third, 10 M nitric acid is added into the gel to cause AMP precipitation in the silica gel pores, after which the gel is aged for 24 h. Finally, the solvent is extracted from the gel pores at ambient conditions, forming the AMP-silica nanocomposites.

#### 2.2.2. Synthesis of Functional Silica-Based Nanoparticle with Metal Molybdophosphoric via an Impregnation Method

A two-step impregnation method is adopted to prepare highly dispersed metal molybdophosphoric onto silica-based nanoparticles [32]. Generally, in the first step, molybdophosphoric acid is placed into an aqueous solution containing 40% to achieve ethanol incipient wetness impregnation into silica-based nanoparticles, after which it is dried at 100 °C and calcined at 300 °C for 3 h. In the second step, a metal-salt is impregnated into an aqueous solution containing 0.3 M HCl and is mixed with silica-based nanoparticles modified with molybdo-phosphoric. Finally, the samples are refluxed at 85–90 °C for 24 h, after which the sample was filtered, washed with demineralized water, and dried in an oven at 50 °C for 24 h. Advantages of the materials synthesized by the wet impregnation method are that they possess the mesostructure and high thermal stability of the silica-based nanoparticle host, as well as the effective ion exchange properties of the dispersed Tin (IV) molybdophosphate. Furthermore, this surface impregnation method allows better control over the homogeneity of the Tin (IV) molybdophosphate.

#### 2.2.3. Adsorption Performance of Ion Sorption by Silica-Based Nanoparticles Modified with Metal Molybdophosphate

According to a study of the titanium (IV) molybdophosphateions (TMP) in the sorption of various metal ions, TMP has a high affinity for Cs^+^, Sr^2+^, UO_2_^2+^, Ba^2+^, Pb^2+^, Tl^+^, Zn^2+^, Rb^2+^, and Zr^4+^ ions [99]. Separation of metal ions on titamium (IV) molybdophosphate at room temperature is listed in Table 6. The properties of TMP for the adsorption of different metal ions are based on the value of the distribution coefficients (Kd), which are obtained through experiments in demineralized water and nitric acid media. According to Reference [100], in addition to the intrinsic property of the adsorbent, factors such as the solvent distribution, nature of the chemical bond, and formation of complexes, also affect the wide variation in the distribution coefficient values. Based on the values of Kd, the affinity of TMP for the sorption of alkali metal ions is in the following order: Cs^+^ > Rb^+^ > K^+^ > Na^+^ > Li^+^. This may be due to the size of the hydrated radii of the exchanging ions, based on which ions with small radii could easily enter the pores of the adsorbent [52,53]. However, for most metal ions, the value of Kd decreases with the increase in concentration of nitric acid. The competitive adsorption on the sites of the adsorbent between the metal ions and H_3_O^+^ ions may result in low Kd values of the metal ions at high concentrations of acid. The experimental results suggest that TMP has a greater affinity for ions such as Cs^+^, Sr^2+^, UO_2_^2+^, Ba^2+^, Pb^2+^, Tl^+^, Zn^2+^, Rb^2+^, and Zr^4+^ than other metal ions, which indicates that TMP could selectively remove those ions from complex systems. Furthermore, the adsorption of ions could be applied for systems containing nuclear waste, alloys, minerals, and heavy metals/trace metals, and even for the separating special radioactive ions for microlevel quantitative analysis. The silica-based nanoparticles modified with TMP extend the applications of this type of materials.

#### 2.2.4. Specific Retention Mechanism of Ion Sorption

According to Reference [101], the phosphomolybdate complex ion (PMo_12_O_40_)^3−^ consists of 12 MoO_6_ octahedra, with a PO_4_ group in the center of the crystal structure of the metal salt, which forms a hollow sphere. The hollow sphere has a structure containing pores and tunnels [102]. The metal ions with associated water molecules fit between the spheres of negative ions in the pores and tunnels. For the adsorption of Cs^+^ on ammonium molybdophosphate (AMP), the uptake of Cs^+^ ions in AMP occurs due to isomorphous exchange of Cs^+^ ions for NH_4_^+^ ions in the crystal lattice [103]. The exchange of Cs^+^ ions is due to the small size of the “hydration sphere” of Cs^+^ ions. Nevertheless, larger hydrated ionic radius ions cannot bind to the deeply seated negative charges in the AMP matrix. For example, the ^137m^Ba^2+^ species elutes quickly due to the open porous structure of AMP, which favors facile release of this ion by diffusion.

### 2.3. Silica-Based Nanoparticle Modified with Titanate-Based Materials

**Figure 4:**
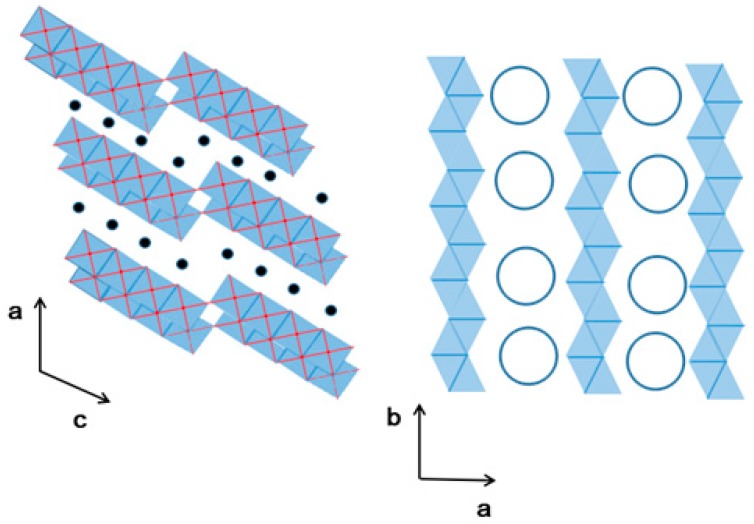
The structure modes of K_2_Ti_6_O_13_ viewing along the {0 1 0} direction.

Titanate has a layered perovskite structure, and the octahedral structure of TiO_6_ forms a perovskite layer by sharing angles, edges, and surfaces [104,105]. The structure modes of K_2_Ti_6_O_13_ viewing along the {0 1 0} direction is shown in Figure 4. Nanosized crystalline powders can be synthesized through traditional approaches, such as the sol-gel, hydrothermal, and solid-phase methods. Obviously, such nanoscale crystalline powders cannot be used in industrial separation columns for the continuous treatment of wastewater. To solve this problem, companies such as Japan Kurita Industries adopted a method in which titanium acid salt and clay are mixed to prepare large granular adsorbent titanate crystals. This process not only improves the mechanical strength of the adsorbent but also allows the material to be used in large-scale industrial adsorption towers. However, the specific surface area of the adsorbent is small, and the adsorption rate is slow, which is attributed to larger adsorbent particles, higher particle agglomeration pressure, and higher density. Such disadvantages cannot be ignored in industrial processing systems. Loading titanate onto a new type of porous silica-based microsphere was recently developed by Wei and his team. The synthesized silicon matrix composites are easily loaded and unloaded from the separation column, and have low column pressures after loading. The advantages of silicon matrix carrier materials are fully reflected especially when used in an industrial-scale adsorption column separation process. Preparation of new high-efficiency K_2_Ti_6_O_13_/SiO_2_ adsorbents by loading titanate onto a SiO_2_ matrix material is promising for effectively removing Sr^2+^ in radioactive pollution wastewater due to accidents with high concentrations of salt.

#### 2.3.1. Synthesis Procedures

Silica-based nanoparticles modified with K_2_Ti_6_O_13_ are synthesized using the sol-gel method [54,55]. A certain mass of SiO_2_-F50 is cleaned with methanol three times, for 30 min each time. After cleaning, the material is moved into a vacuum drying oven at 80 °C. Tetrabutyl titanate and potassium acetic acid are added according to their stoichiometric ratios, and dissolved in ethylene glycol methyl ether and glacial acetic acid, respectively. The cleaned SiO_2_-F50 powder is added and stirred for 30 min to dissolve. The sol mixture is transferred into a rotating flask and heated continuously at a temperature of 50 °C. The cooling circulating water and vacuum pump equipment is opened, and the organic solvent in the sol is allowed to evaporate. The rotary flask is removed and dried in a vacuum drying box at 80 °C. The dried gel powder is placed in a high-temperature sintering furnace and annealed at various temperatures.

**Figure 5:**
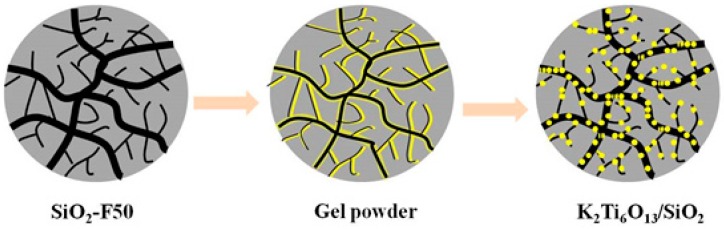
Process of K_2_Ti_6_O_13_/SiO_2_ preparation [48].

The synthesis process aims to immobilize sol-gel molecules into the pores of the SiO_2_-F50 substrate through rotatory evaporation through the sol-gel method. The dry gel powder was sintered at high temperatures to cause potassium hexatotate crystals to grow in the pores of SiO_2_-F50 and form K_2_Ti_6_O_13_/SiO_2_ inorganic composite material. The preparation and formation process of the material is shown in Figure 5.

#### 2.3.2. Adsorption Performance of Ion Sorption by Silica-Based Nanoparticles Modified with Titanate

The selectivity of K_2_Ti_6_O_13_/SiO_2_ for cationic exchange has the following order: Sr^2+^ > Ca^2+^ > Cs^+^ > Mg^2+^ > Na^+^. In addition, the large numbers of co-existing Ca^2+^ and Na^+^ ions have a great impact on the adsorption of Sr^2+^. The adsorbent can effectively remove Sr^2+^ from accident wastewater, but it is not suitable to directly treat radioactive wastewater systems, such as seawater [48]. The presence of abundant sodium and potassium in seawater decreases the distribution coefficient values, which leads to competition between the various inorganic cations: a higher concentration of ions corresponds to a smaller distribution coefficient [106]. According to a previous report [48], the adsorption treatment of Sr^2+^ by the K_2_Ti_6_O_13_/SiO_2_ adsorbent in simulated Fukushima nuclear accident contaminated wastewater was also carried out. The adsorption capacity at the penetration point of 5% was calculated by changing the flow rate and column diameter of the fluid. The experimental results show that the slower flow rate and longer column diameter effectively improved the adsorption capacity for the wastewater. According to the penetration curve, the saturated adsorption capacity of the adsorbent was 15.2 mg/g, and the column utilization rate was 83%.

#### 2.3.3. Specific Retention Mechanism of Ion Sorption

Generally, a layered structure is much less stable than a three-dimensional lattice structure, and cations in the layered titanate structure can easily produce ion-exchange reactions with specific cations in the solution [107,108,109,110]. The adsorption of Sr^2+^ for titanate occurs through an ion-exchange reaction. Formulas (1) and (2) shows the reaction of the ion exchange between titanate and Sr^2+^. To explore the essence of the ion exchange reaction between the adsorbent of silica-based nanoparticles modified by titanate and Sr^2+^, SEM and EDS analyses were used in a previous report [111]. The results suggest that K_2_Ti_6_O_13_/SiO_2_ reacts with Sr^2+^ in solution through ion exchange, and the mechanism of the ion-exchange can be expressed as:

(1) $$K_{2}{\mathrm{Ti}}_{n}O_{2n+1}+{\mathrm{Sr}}^{2+}⇌{\mathrm{SrTi}}_{n}O_{2n+1}+2K^{+}$$

(2) $$K_{2}{\mathrm{Ti}}_{6}O_{2}/S_{i}O_{2}+{\mathrm{Sr}}^{2+}⇌{\mathrm{SrT}}_{i}O_{13}/{\mathrm{SiO}}_{2}+2K^{+}$$

### 2.4. Hydrotalcite-Modified Silica-Based Microparticle

**Figure 6:**
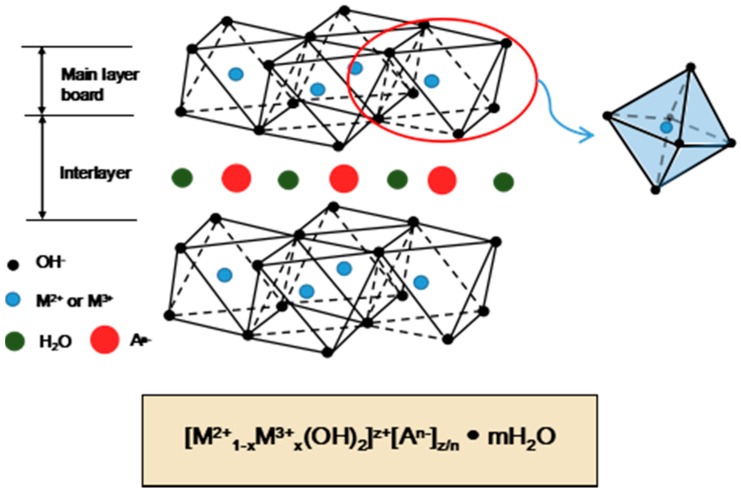
The basic structure of hydrotalcite.

Layered double hydroxides (LDHs) and their modified forms are recognized as efficient adsorbents for the removal of numerous ionic contaminants from aqueous solutions, such as UO_2_^2+^ [111,112], ^241^Am^3+^ [112], Cu^2+^, Pb^2+^, and Cr^6+^ [113]. LDHs possess the important property of a restoration (or memory) effect of their layered structures, which can be described as follows: during calcination at 300–500 °C, the water and original anions in the interlayers of the LDHs can be eliminated and form layered double oxides (LDOs) [114,115,116]; the restoration reaction of the lamellar structure occurs when the LDOs are exposed to solutions with additional anions, which act as charge-balancing anions, spontaneously inserting into the interlayers. These reconstituted LDHs may be distinguished from their original structures by the interlayer incorporation of different anions. The above-mentioned structure memory effect has been successfully applied to increase the adsorption capacity of LHDs for anions. Many of the disadvantages of general inorganic adsorbents are also present in LDHs. Loading LDHs onto a suitable substrate can not only significantly reduce disadvantages such as nanoparticle aggregation, but also can greatly improve abrasion resistances, mechanical endurances, and the hydraulic performances of LDHs. Furthermore, the porous silica spheres can also maintain good porous microstructures and morphologies during the transformation of the LDHs to LDOs by calcination at high temperatures [33]. The basic structure of hydrotalcite is presented in Figure 6.

#### 2.4.1. Synthesis Procedures

**Figure 7:**
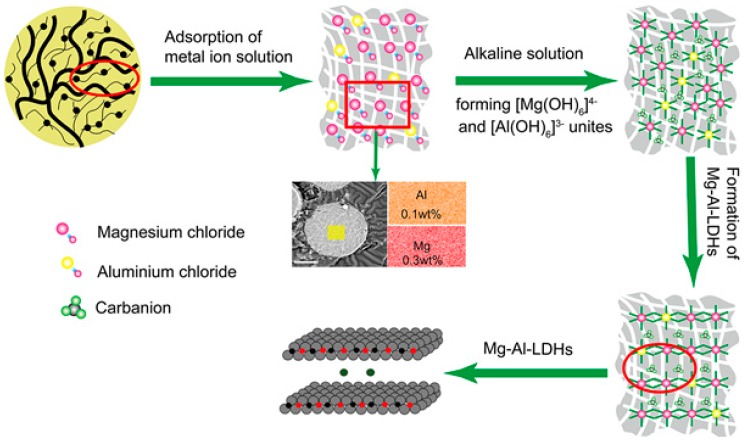
The synthesis mechanism diagram of Mg-Al-Hydrotalcite in the channel of silica matrix [33].

The preparation process of LDHs-SiO_2_ is given as follows: Step 1: AlCl_3_•6HO_2_ and MgCl_2_•6HO_2_ (molecular proportion of Mg and Al = 3:1) are dissolved into deionized water to form a mixed metal ion solution. Step 2: SiO_2_ powder is placed into the mixed solution, and the solution is subsequently moved into a vacuum rotary evaporator for 6 h to ensure that the mixed metal ions fill into the channels of the SiO_2_ powder. Step 3: A caustic solution is prepared by dissolving NaCO_3_ and NaOH (mass ratio = 2:1) into deionized water. The solution prepared in Step 2 is added dropwise to the caustic solution through a long-stem funnel, and the pH is kept above 10 by adding NaOH during the reaction process. Mg-Al-LDH/SiO_2_ powder is obtained after vacuum filtration of the reacted solution, washing with a 0.1 M NaCO_3_ solution three times, and drying at 80 °C for 4 h. Step 4: The obtained Mg-Al-LDH/SiO_2_ powder is calcined at 450 °C to form a Mg-Al-LOD/SiO_2_ composite [33]. The synthesis mechanism diagram of Mg-Al-Hydrotalcite in the channel of silica matrix is presented in Figure 7.

#### 2.4.2. Adsorption Performance of Ion Sorption by Silica-Based Nanoparticles Modified with Hydrotalcite

The thermal treatment of hydrotalcite greatly affects the sorption of I^−^. In the calcined samples the sorption significantly increases, showing that I^−^ is not able to easily replace the anions in the hydrotalcites. However, when some anions are removed by a calcination process producing oxides, I^−^ ions can occupy the anionic sites when the hydrotalcites crystallize again following aqueous solution addition. According to a previous report [33], Mg-Al-LDO/SiO_2_ exhibited excellent adsorption performance for I^−^, and the removal efficiency reached 99.81% in a 30 mg/L I^−^ solution in 5 min using a dosage of 0.05 g/100 mL.

#### 2.4.3. Specific Retention Mechanism of Ion Sorption

Typically, partial divalent cations (M^2+^) in the lamellar structure of the LDHs are substituted by isomorphous trivalent cations (M^3+^), resulting in a net permanent positive charge in the trioctahedral position of the hydroxide layers, which is balanced by exchangeable anions intercalated into the interlayers. Depending on the types of metal cations in the layered structure and exchangeable anions present in the interlayers, LDHs can exhibit adsorption properties for various ions [117].

## 3. Porous Silica-Based Nanoparticles Modified by Organic Materials

The adsorption of typical fission products and some other actinides, using organic supramolecular recognition materials, has drawn considerable attention. Many studies on the separation and removal of typical fission products and rare earth elements, by loading different organic functional groups onto silica-based spheres, have been carried out by Wei and his team. The main functional groups and the specific adsorption of ions are listed in Table 2. We summarize the general procedure for obtaining these composites, the specific adsorption for different ions, and possible mechanisms of adsorption in this section.

### 3.1. General Synthesis Process of Functional Silica-Based Nanoparticles Modified by Organic Materials

The synthesis method for SiO_2_-P modified with different organic functional groups can be generalized as follows: (1) the abstersion and activation of the substrate of SiO_2_-P. SiO_2_-P is added into a 500 mL conical flask, and 200 mL methanol is slowly added to the flask, which is vibrated for 30 min in a water bath. Subsequently, the suspension liquid is filtered using a vacuum suction filter device, and the above process is repeated three times. The SiO_2_-P vacuum dried at 313 K for 24 h. The washing and activation process are mainly used to improve the affinity between organic macromolecular materials and molecular polymers in SiO_2_-P; (2) functional organic materials are dissolved into 200 mL of organic solvent and strongly stirred for 30 min. The organic materials are completely dissolved into the solutions, and the mixed organic solution is transferred to the flask. The water bath is set to 298 K. The speed of the rotary evaporation apparatus is slowly increased to the maximum, and the vacuum pump is opened after rotating the flask for 4 h. The water bath temperature is slowly increased to 315 K to dry the organic solvent; and (3) the large flask containing the drying solvent is placed in a vacuum drying oven, and the drying temperature is set to 315 K. After drying for 24 h, the synthesized adsorbent is bottled and set aside. The above-mentioned steps are based on two previous reports [118,119].

### 3.2. Specific Ion Sorption by Functional Silica-Based Nanoparticles Modified with Various Organic Groups

#### 3.2.1. Silica-Based Calix {4} Arene-R14 Adsorbent

**Figure 8:**
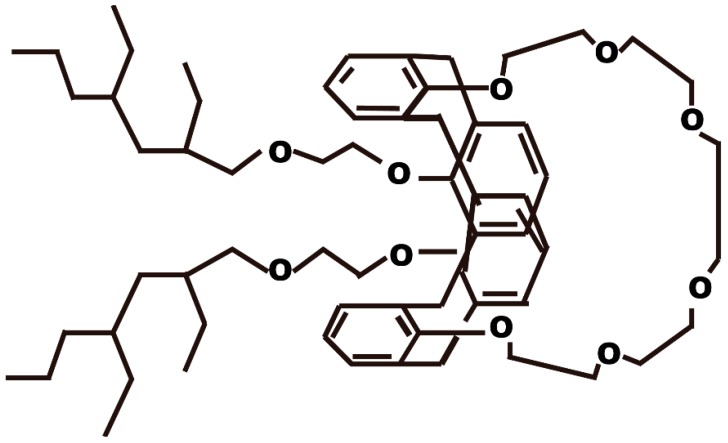
The chemical structural formula of Calix {4} arene-R14 [56].

**Figure 9:**
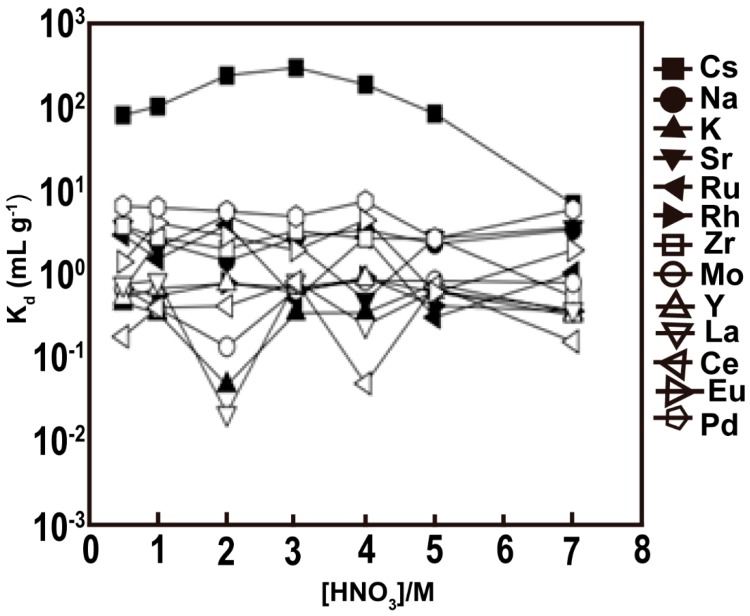
Dependence of Kd of tested metals on (Calix {4} + dodecanol)/SiO_2_-P. [metal] = 10 ppm; temperature = 298K; and phase ratio = 0.05 g /10 cm^3^ [56].

Calix {4} arene-R14, as a macromolecular organic extraction agent, has excellent selective extraction performance for Cs^+^ ions in nitric acid, and its structural formula is shown in Figure 8. The silica-based Calix {4} arene-R14 adsorbent exhibits the best adsorption performance for Cs^+^ in a 3 M nitric acid solution, and can be applied for the treatment of Cs^+^ in a highly radioactive waste solution. To evaluate the selectivity properties for Sr^2+^ ions, the adsorption behavior was evaluated by adsorbing strontium ions in a mixed solution containing various ions, including Cs^+^, Na^+^, K^+^, Sr^2+^, Pd^2+^, Ru^3+^, Rh^3+^, Zr^4+^, Mo^5+^, Y^3+^, Ce^3+^, La^3+^, and Eu^3+^. The experimental results (Figure 9) showed that the distribution coefficient of Cs^+^ increased with increasing concentrations of nitric acid when the concentration of nitric acid was 0.5–3 M, and the maximum distribution coefficient was 300 cm^3^/g when the concentration of nitric acid was 3 M. Nevertheless, when the concentration of nitric acid was between 3 and 7 M, the distribution coefficient of Cs^+^ decreased with increasing nitric acid concentrations. Calix {4} arene-R14 has a crown ether ring hole size of 0.162 nm, which is very close to that of Cs^+^ ions of 0.167 nm, and nitrates were used as coordination ions [56]. When the concentration of nitric acid was greater than 3 M, the decrease in the distribution coefficient of Cs^+^ may be due to the interaction between Calix {4} arene-R14 and nitric acid molecules. Silica-based Calix {4} arene-R14 adsorbents do not significantly adsorb other metal ions when the concentration of nitric acid is within the range of 0.5–7 M, indicating that the adsorbents had very good selectivity for Cs^+^ ions.

#### 3.2.2. BTP/SiO_2_-P Adsorbents

BTPs (BTP: 2,6-bis-(5,6-dialkyl-1,2,4-triazin-3-yl) -pyridines) can be as environmentally friendly as N-donor molecules. Recently, BTPs were used to separate MA(III) from high-level radioactive waste liquid by Kolarik [54,55]. BTPs have received wide attention due to their high selectivity of MA(III) over Ln (III) from nitrate solutions. BTPs have been developed by modifications with branched alkyl groups, such isopropyl-BTP, isobutyl-BTP, and isohexyl-BTP [120,121], in addition to BTPs with cyclic structures, e.g., CA-BTP, CyMe_4_-BTP, and Me_2_-CA-BTP [60,122,123], which have been designed and synthesized for the purpose of improving the stability. Based on BTPs’ solubilities, Wei et al. proposed and designed a compact and effective chromatographic extraction process using a BTP/SiO_2_-P adsorbent to separate MA(III) from High-level radioactive waste liquid (HLLW). We summarize the specific adsorption of ions onto the adsorbent of BTP/SiO_2_-P modified with different branched alkyl groups below.

#### 3.2.3. Adsorption of MA (III) and Ln (III), Minor Actinides on Me_2_-CA-BTP/SiO_2_-P Adsorbent

**Figure 10:**
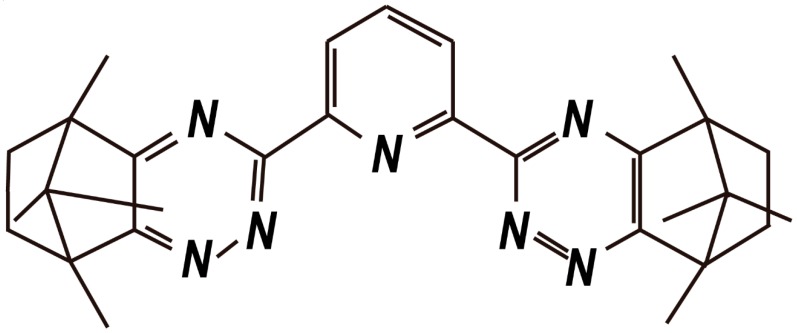
Structure formula of Me_2_-CA-BTP (5,6,7,8-tetrahydro-5,8,9,9-tetramethyl-5,8-methano- 1,2,4-benzotriazing-3-yl) pyridine) [57].

**Figure 11:**
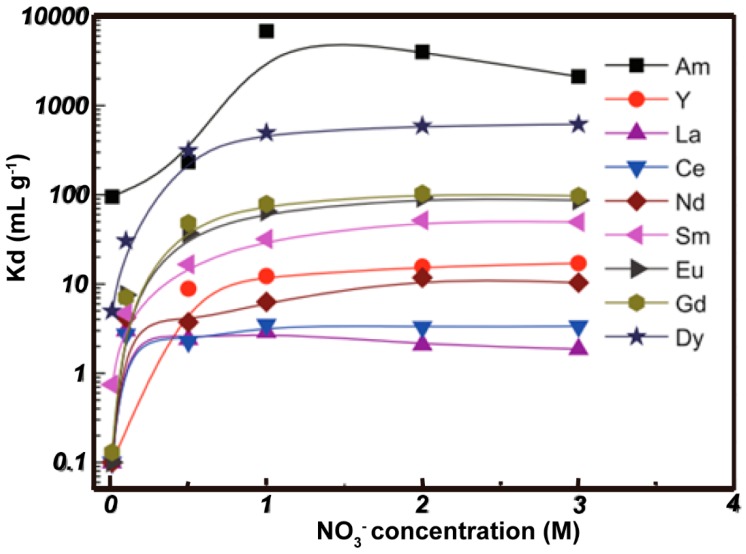
Effect of nitrate concentration on adsorption (0.01M HNO_3_; phase ratio = 0.05 g/2.5 mL; ^241^Am (III) = 1000 Bq/mL; Ln (III) = 1 mM; temperature = 298 K; contact time = 24 h; and sharking speed = 120 r/min) [57].

Me_2_-CA-BTP (Figure 10), namely, (2,6-bis (5,6,7,8-tetrahydro-5,8,9,9-tetramethy l-5,8-methano- 1,2,4-ben zotriazin-3-yl) pyridine), is an efficient adsorbent for the adsorption of MA (III) and Ln (III). The Me_2_-CA-BTP/SiO_2_-P adsorbent has a strong affinity for Am (III) over Ln (III) fission products with high adsorption and selectivity properties, and separation factors (SF) reaching 557, 2355, 1952, 1082, 214, 105, 86, and 14 for Y, La, Ce, Nd, Sm, Eu, Gd, and Dy, respectively, in a 0.01 M HNO_3_-0.99 M NaNO_3_ solution. Moreover, the Me_2_-CA-BTP/SiO_2_-P adsorbent exhibited a higher adsorption affinity for Dy (III) than for Eu (III), which explains the higher distribution coefficients of Dy (III) than those of Eu (III) as the nitrate concentration changed [57]. The results are presented in Figure 11.

#### 3.2.4. Adsorption of Am (III), Ln (III), and Dy (III) on IsoHex-BTP/SiO_2_-P Adsorbent

**Figure 12:**
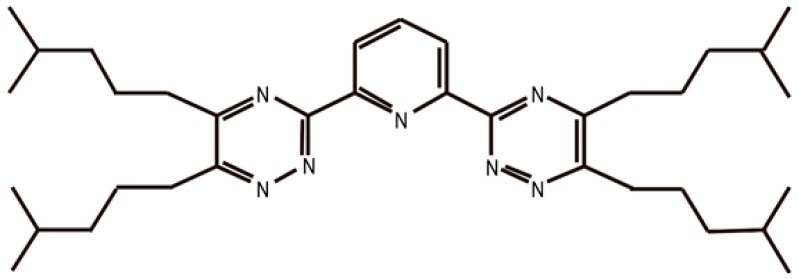
The chemical structure of isoHex-BTP [58].

**Figure 13:**
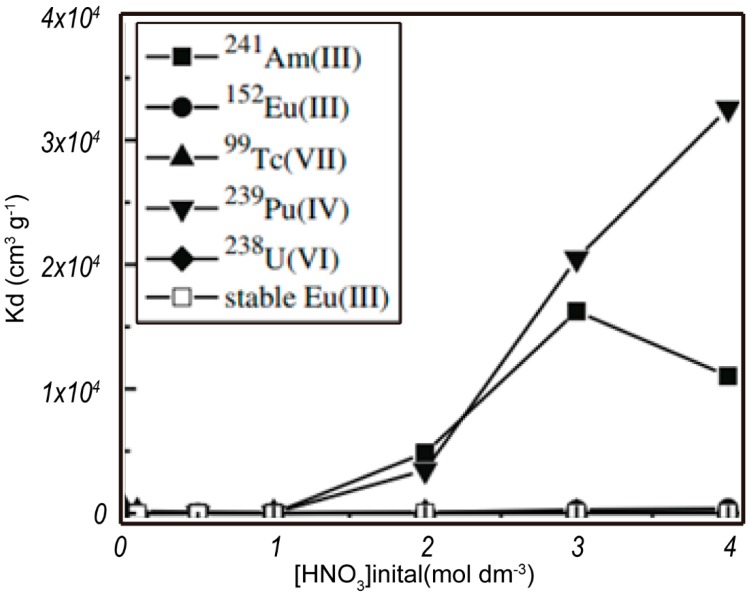
Effect of initial nitric acid concentration on the distribution coefficient of ^241^Am(III), ^239^Eu(III), ^239^Pu(IV), ^99^Tc (VII), ^238^U(VI), and stable Eu(III) (298 K; phase ratio = 0.1 g; 5 cm^−1^, trace of ^241^Am(III), ^239^Eu(III), ^239^Pu(IV), ^99^Tc (VII), ^238^U(VI), and stable Eu(III); 1 mmol dm^−3^; sharking speed = 120 rpm; contact time = 24 h) [58].

The isoHex-BTP is 2,6-bis (5,6-diisohexyl)-1,2,4-triazin-3-yl) pyridine and the chemical structure are shown in Figure 12. Silica-based nanoparticles modified with isoHex-BTP exhibited high affinities and selectivities for Am (III) and Pu (IV) over U(VI), Ln (III), and other typical fission products in 3 mol dm^−3^ nitric acid, as shown in Figure 13. The adsorption data as a function of contact time fit much better to a pseudo-second-order kinetic model with high correlation coefficients (0.988 < R^2^ < 1.000) than to a pseudo-first-order kinetic model, indicating that Dy (III) adsorption by isoHexBTP/SiO_2_-P occurs by a chemisorption mechanism, and that the coordination reaction between isoHex-BTP and Dy (III) is the rate-controlling step of the adsorption process. The adsorption data, depending on the initial Dy (III) concentration, were analyzed by the Freundlich and Langmuir isotherm models. The equilibrium adsorption data followed the Langmuir isotherm model more closely than the Freundlich isotherm model at temperatures of 288, 298, and 308 K, which indicates that the adsorption of Dy (III) occurs on a homogeneous surface of isoHex-BTP/SiO_2_-P, and that each adsorptive site of isoHex-BTP/SiO_2_-P can be occupied by Dy (III) only once in a one-on-one manner [58].

#### 3.2.5. Adsorption of ^241^Am (III) over Y (III) and Ln (III) on Silica/Polymer-Based Isobutyl-BTP/ SiO_2_-P Adsorbent

**Figure 14:**
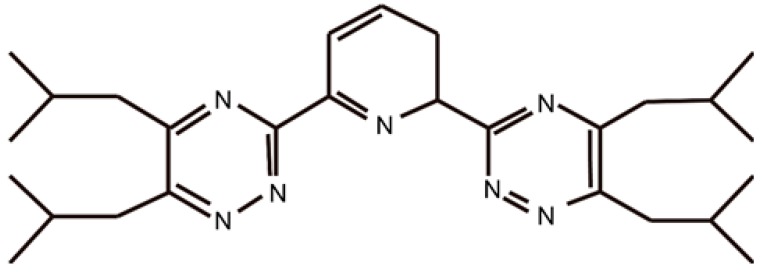
Chemical structure of isobutyl-BTP(2,6-diisobutyl-1,2,4-triazin-3-yl) pyridine) [59].

**Figure 15:**
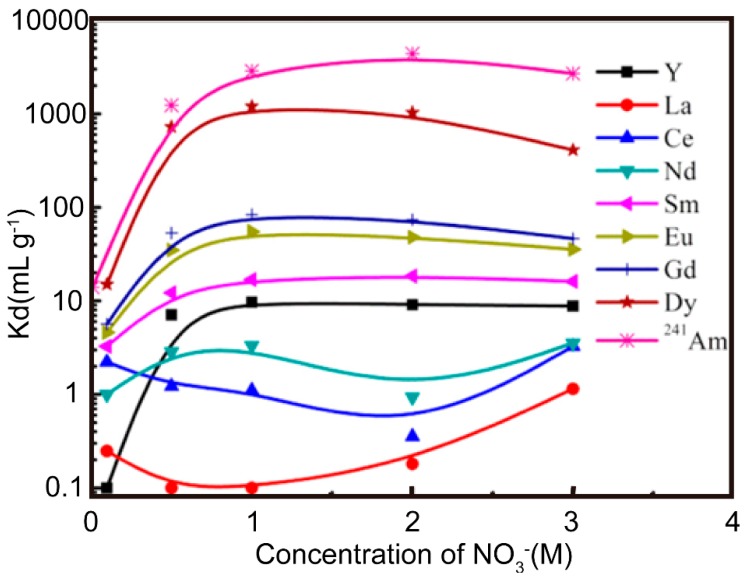
Effect of nitrate concentration on adsorption (0.01 M HNO_3_; phase ratio = 0.1g/5 mL; ^241^Am (III) = 1000 Bq mL^−1^; Ln (II) = 1 mM; temperature = 298 K; contact time = 24 h; speed = 120 rpm) [59].

Isobutyl-BTP/SiO_2_-P (Figure 14) shows strong adsorption and high selectivity for ^241^Am (III) over Ln (III) fission products in a wide nitrate concentration range of 0.5–3.0 M, with the separation factor reaching dozens, hundreds, or more. Figure 15 indicates that it is a promising adsorbent candidate for the separation of MA (III) from Ln (III) in high-level radioactive waste liquid. Dy (III) can be regarded as a simulated element of MA(III). Its adsorption kinetics were well-captured by a pseudo-second-order rate model, and the adsorption isotherm data matched well with the Langmuir isotherm adsorption model [59].

### 3.3. Adsorption of Minor Actinides on Silica/Polymer-Based CA-BTP Adsorbent

**Figure 16:**
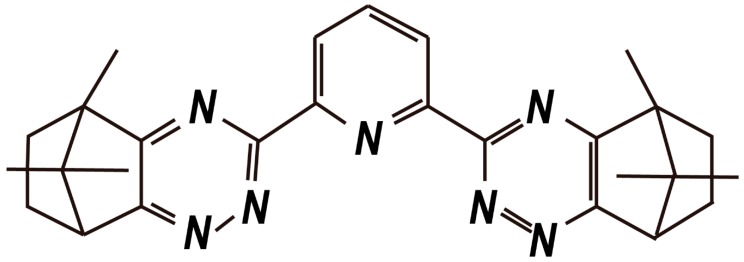
Chemical structure of CA-BTP (bis-2,6-(5,6,7,8-tetrahydro-5,9,9-trimethyl-5,8-methano -1,2,4-benzotriazing-3-yl) pyridine) [60].

**Figure 17:**
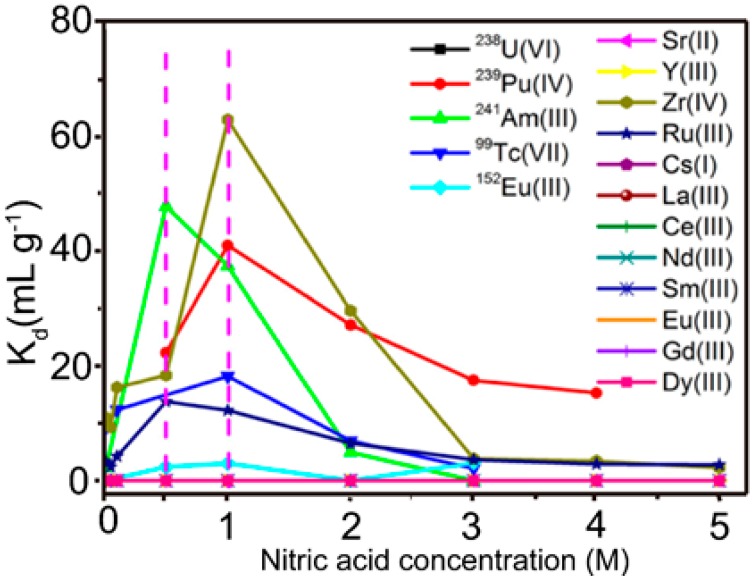
Effect of initial nitric acid concentration on adsorption (adsorbent 0.1 g; solution = 5 mL; ^241^Am(III), ^152^Eu (III), and ^99^Tc(III) = 1000 Bq mL^−1^; ^239^Pu(IV) = 50 Bq mL^−1^; ^238^U (VI) = 1 mM. The other method ions: 1 mM; temperature = 25 °C; contact time = 24 h; speed = 120 rpm) [60].

The CA-BTP is bis-2,6-(5,6,7,8-tetrahydro-5,9,9-trimethyl-5,8-methano-1,2,4-benzotriazing-3-yl) pyridine) and its chemical structure is shown in Figure 16. The adsorption properties of CA-BTP/SiO_2_-P for ^238^U(VI), ^239^ Pu (IV), ^241^Am (III), ^99^Tc (VII), ^152^Eu (III), and some typical fission products were studied, and the results are presented in Figure 17. The CA-BTP/SiO_2_-P stability against c-radiation was also evaluated. CA-BTP/SiO_2_-P showed very poor adsorption abilities toward U(VI) and most experimental FPs (where FP = Sr(II), Zr(IV), Cs(I)), while CA-BTP/SiO_2_-P exhibited higher adsorption abilities toward ^241^Am (III), ^239^ Pu (IV), and ^99^Tc (VII) in a 0.5 M HNO_3_ solution. Moreover, dry CA-BTP/SiO_2_-P demonstrated no instability when the radiation dose was up to 161 kGy. The CA-BTP/SiO_2_-P adsorbent is a potential candidate for separating ^241^Am (III), ^239^Pu (IV), and ^99^Tc (VII) from high-level radioactive waste liquid [60].

## 4. Concluding Remarks

The functional silica-based nano/micro-particles modified with various organic or inorganic materials are prepared by various synthesis methods. Generally, there are more modification choices for loading inorganic materials onto silica-based substrates, such as the direct synthesis method, wetness impregnation method, and layer-by-layer (LBL) deposition method, than for organic groups, which are mainly based on the wetness impregnation method. By summarizing the results of the adsorption experiments, it can be concluded that the introduction of silica-based substrates does not change the adsorption properties of the loaded material for the target ions. On the contrary, introduction of the silicon-based carrier only changes the mechanical characteristics of the materials, such as abrasive resistance, dispersibility, and radiation resistance, which greatly improve the capacity of the adsorption for target ions. Notably, silica-based substrates significantly improve the irradiation resistance of the modified adsorbent, especially for the organic-modified materials that are usually used for the separation of radioactive radicals. Although there has been considerable progress in the development of organic adsorbents, these materials still do not meet the practical requirements. From a chemical perspective, the introduction of silica-based substrates has solved two major problems. One problem is the formation method of the chemical materials, which is necessary so that the adsorption properties of the adsorbent in exchange columns are retained, or to accommodate with a large number of solutions when the powder adsorbent is made into a specified shape. Another problem is the disadvantage of inorganic ion exchange materials for improving the chemical stability of the framework, which determines the cycle stability of the adsorption and desorption processes. These are the general conclusions drawn from this review on functional silica-based nano/micro-particles.

## Figures and Tables

**Table 1:** Structural properties of the ordered mesoporous silica-based carriers.

Sample	Surface Area (m^2^ g^−1^)	Particle Size a_0_ (nm)	Pore Volume (cm^−3^ g^−1^)	Pore Diameter (nm)	Reference
SBA-15	773	12.1	1.16	7.8	[6]
MCM-41	1268	4.196	0.92	2.5	[2]
MCM-48	923	50	0.63	3.8	[3]
M41S	1064	4.196	0.942	3.54	[4]
SBA-1	475	11.18	1.09	3.0	[5]
FSM-16	1002	4.3	0.969	3.9	[7]
Silica Sphere	640	75–150 um	0.29	2.8	[8]

**Table 2:** The silica-based materials modified by the main inorganic materials.

Silica-Based Substrate	Inorganic Materials	Loading Methods	Specific Sorption of Reference	Reference
SBA-15	Metal phosphates	Wetness impregnation, Layer-by-layer (LBL) deposition approach	Ni^2+^, Hg^2+^, Pb^2+^	[31,32,49,50,51]
SBA-15	Metal molybdophosphate	Impregnation method	Cs^+^, Sr^2+^, UO_2_^2+^, Ba^2+^, Pb^2+^, Tl^+^, Zn^2+^, Rb^2+^, Zr^4+^	[52,53]
Silica sphere	Titanate based materials	Surface sol-gel method	Sr^2+^	[54,55]
Silica sphere	Hydrotalcite	Impregnation method	I^−^	[32]

**Table 3:** The SiO_2_-P adsorbents modified with the specific organic macromolecules, and their applications for treating various ions.

Silica-Based Substrate	Organic Materials	Loading Methods	Specific Sorption of Ions	Reference
Silica sphere	Calix [4] arene-R14	Impregnation method	Cs^+^	[54,56]
Silica sphere	Me_2_-CA-BTP	Impregnation method	MA (III) and Ln (III)	[57]
Silica sphere	isoHex-BTP	Impregnation method	Am (III) and Pu (IV)	[58]
Silica sphere	isobutyl-BTP	Impregnation method	^241^Am (III) over Y (III) and Ln (III)	[59]
Silica sphere	CA-BTP	Impregnation method	^241^Am (III), ^239^ Pu (IV), and ^99^Tc (VII)	[60]

**Table 4:** Adsorption percentage of metal ions on the lanthanum phosphate in aqueous media.

Adsorbent	Adsorption Percent/w%										
	Cr (III)	Mn (III)	Fe (III)	Co (III)	Ni (III)	Cu (III)	Zn (III)	Cd (III)	Ba (III)	Hg (III)	Pb (III)
Uncalcined sample	4.06	10.52	16.54	8.08	99.97	9.27	22	14.58	1.90	99.99	30.70
Calcined sample	6.91	7.14	15.07	8.45	12.43	8.94	23.65	15.07	3.04	41.12	31.97

**Table 5:** Adsorption percentage of metal ions on the lanthanum phosphate in 3 mol L^−1^ of ammonia.

Adsorbent	Adsorption Percent/w%										
	Cr (III)	Mn (III)	Fe (III)	Co (III)	Ni (III)	Cu (III)	Zn (III)	Cd (III)	Ba (III)	Hg (III)	Pb (III)
Uncalcined sample	98.25	99.82	99.96	58.3	12.51	21.99	88.32	54.43	29.97	92.42	99.99
Calcined sample	97.51	99.96	99.98	57.9	15.57	35.37	89.20	30.20	78.80	73.88	99.94

**Table 6:** Separation of metal ions on titamium (IV) molybdophosphate at room temperature.

No.	Metal Separated (ug)	Amount Loaded (ug)	Amount Found (ug)	Total Elution Volume (mL)	Element Used
1	La	350	350	35	H_2_O
	Ce	450	410	40	0.2 mol^−1^ HNO_3_
2	Mo	720	720	35	H_2_O
	Zr	240	240	30	0.5 mol^−1^ HNO_3_
3	Nd	1080	1050	45	H_2_O
	Ce	400	390	35	0.1 mol^−1^ HNO_3_
4	Bi	1568	1490	50	0.1 mol^−1^ HNO_3_
	Zn	200	190	40	0.5 mol^−1^ HNO_3_
5	Mo	960	960	50	H_2_O
	Pb	522	505	25	0.5 mol^−1^ HNO_3_
6	Li	105	105	20	H_2_O
	K	98	90	25	0.2 mol^−1^ HNO_3_
7	Dy	1625	1550	45	H_2_O
	Ce	400	380	30	0.2 mol^−1^ HNO_3_
8	Y	1000	950	60	H_2_O
	Ti	510	500	45	0.5 mol^−1^ HNO_3_
9	Li	140	140	20	H_2_O
	Rb	440	440	30	0.2 mol^−1^ HNO_3_
10	Mo	1000	990	55	H_2_O
	Cs	600	660	25	0.2 mol^−1^ HNO_3_
11	Mo	1000	990	20	H_2_O
	Sr	400	430	20	0.1 mol^−1^ HNO_3_
